# Brief electrical stimulation after facial nerve transection and neurorrhaphy: a randomized prospective animal study

**DOI:** 10.1186/s40463-016-0118-6

**Published:** 2016-02-01

**Authors:** Adrian Mendez, Hadi Seikaly, Vincent L. Biron, Lin Fu Zhu, David W. J. Côté

**Affiliations:** Department of Surgery, Division of Otolaryngology-Head and Neck Surgery, University of Alberta, Edmonton, AB Canada; Faculty of Medicine and Dentistry, University of Alberta, Edmonton, AB Canada; 1E4 Walter C Mackenzie Centre, 8440-112 Street NW, Edmonton, AB T6G 2B7 Canada

## Abstract

**Background:**

Recent studies have examined the effects of brief electrical stimulation (BES) on nerve regeneration, with some suggesting that BES accelerates facial nerve recovery. However, the facial nerve outcome measurement in these studies has not been precise or accurate.

The objective of this study is to assess the effect of BES on accelerating facial nerve functional recovery from a transection injury in the rat model.

**Methods:**

A prospective randomized animal study using a rat model was performed. Two groups of 9 rats underwent facial nerve surgery. Both group 1 and 2 underwent facial nerve transection and repair at the main trunk of the nerve, with group 2 additionally receiving BES on post-operative day 0 for 1 h using an implantable stimulation device. Primary outcome was measured using a laser curtain model, which measured amplitude of whisking at 2, 4, and 6 weeks post-operatively.

**Results:**

At week 2, the average amplitude observed for group 1 was 4.4°. Showing a statistically significant improvement over group 1, the group 2 mean was 14.0° at 2 weeks post-operatively (*p* = 0.0004). At week 4, group 1 showed improvement having an average of 9.7°, while group 2 remained relatively unchanged with an average of 12.8°. Group 1 had an average amplitude of 13.63° at 6-weeks from surgery. Group 2 had a similar increase in amplitude with an average of 15.8°. There was no statistically significant difference between the two groups at 4 and 6 weeks after facial nerve surgery.

**Conclusions:**

This is the first study to use an implantable stimulator for serial BES following neurorrhaphy in a validated animal model. Results suggest performing BES after facial nerve transection and neurorrhaphy at the main trunk of the facial nerve is associated with accelerated whisker movement in a rat model compared with a control group.

## Background

Facial neuromuscular disorders and functional impairment resulting from facial nerve injury are common and can be severe [1]. Aesthetic impairments also impart an affliction leading to social isolation and further emotional distress. Together these can lead to depressive symptoms and mental health issues, which further exacerbate their functional disabilities [2]. There are several clinical factors that have been identified that further impact peripheral nerve function recovery following nerve injury including time to repair, type of repair, and the age of the patient [3]. In an effort to optimize recovery, specific repair techniques are utilized that have been shown to improve outcome. The basic requirement is to appose the cut ends of the nerve in such a fashion as to minimize scar formation and preserve the optimal blood supply [4]. In cases of sharp nerve division with minimal gap, direct end-to-end nerve repair is indicated [5]. Tension-free suture repair remains the preferred treatment option as tension will result in scaring and poor regeneration [4, 5].

Despite advances in microsurgical technique, functional recovery following facial nerve transection injury remains suboptimal [6]. Synkinesis, or axonal regeneration from the proximal stump into inappropriate distal pathways, has long been recognized as a significant contributing factor to poor functional recovery [7]. Previous studies have shown that electrical stimulation affects morphological and functional properties of neurons including nerve branching, rate and orientation of neurite growth, rapid sprouting, and guidance during axon regeneration [8, 9]. Specifically, Gordon et al. examined the effect of electrical stimulation on regeneration after nerve transection in a rat sciatic nerve model [6]. The authors were able to demonstrate that electrical stimulation dramatically accelerated both axonal regeneration as well as preferentially re-innervated motor nerves over sensory branches. The authors also found short-term, 1-h periods of stimulation were as effective as long-term stimulation lasting days to weeks [6].

Animal studies have begun to investigate the effects of electrical stimulation on the facial nerve. In 2008, Lal et al. demonstrated that electrical stimulation accelerates facial nerve recovery [1]. In 2012, Foecking et al. confirmed these findings and also demonstrated that single 30-min sessions of stimulation were as effective in improving facial nerve function as prolonged stimulation [10]. However, the outcome model employed by these studies relied on video observation, potentially introducing error.

In 2010, Hadlock et al. studied the effect of electrical stimulation on the facial nerve in a rat model using a precise functional outcomes model capable of detecting micrometer movements of rat whisking [2]. The authors were able to demonstrate improvement in facial nerve functional outcomes in the first 8 weeks. However, the study employed a facial nerve stimulation technique that introduced stimulation prior to nerve injury [2]. In a generalizable clinical setting, this would be less applicable to repair following an unplanned resection or injury.

A recently developed, validated animal model adapted from Heaton et al. was employed to precisely and accurately measure facial nerve function [11]. The objective of this study was to evaluate facial nerve outcomes using BES employed after nerve transection in our validated animal model.

## Methods

### Study design

This prospective randomized control animal trial was conducted at the Surgical Medical Research Institute (SMRI) at the University of Alberta. A previously validated rat facial nerve model was used [11]. Ethics approval was obtained from the Animal Care and Use Committee (ACUC) overseen by the University Animal Policy and Welfare Committee (UAPWC) at the University of Alberta in Edmonton, Alberta [AUP00000785].

### Study subjects

Eighteen female Wistar rats (Charles River Laboratories, Canada) weighing 200–220 g were used for this study. Sample size was calculated based on the study by Heaton et al., which employed a similar outcome measure, powered to detect a difference of 10° in whisking [11]. All rats were housed in pairs at the Health Sciences Laboratory Animal Services (HSLAS) at the University of Alberta. Rats were weighed and handled daily 2 weeks prior to the commencement of the study to reduce animal stress during the study. The 18 rats were block randomized into two groups of 9. Each animal underwent unilateral facial nerve transection and repair at the main trunk of nerve. Group 2 additionally received brief electrical stimulation for 1 h following nerve repair. Facial nerve functional outcome assessment was collected at 2, 4, and 6 weeks post-operatively.

### Facial nerve functional outcome assessment

The facial nerve functional outcome assessment model employed in this study was based on the model previously described and validated by Heaton et al. [11]. This model employs a head fixation device, body restraint, and bilateral photoelectric sensors to detect precise whisker movements as an objective measure for facial nerve function.

#### Head implant

In order to ensure proper head fixation during whisker movement measurement, an implantable head fixation device was required. An animal head implant was bioengineered for this purpose. The implant is composed of acrylic and long threaded screws.

#### Body restraint

**Fig. 1:**
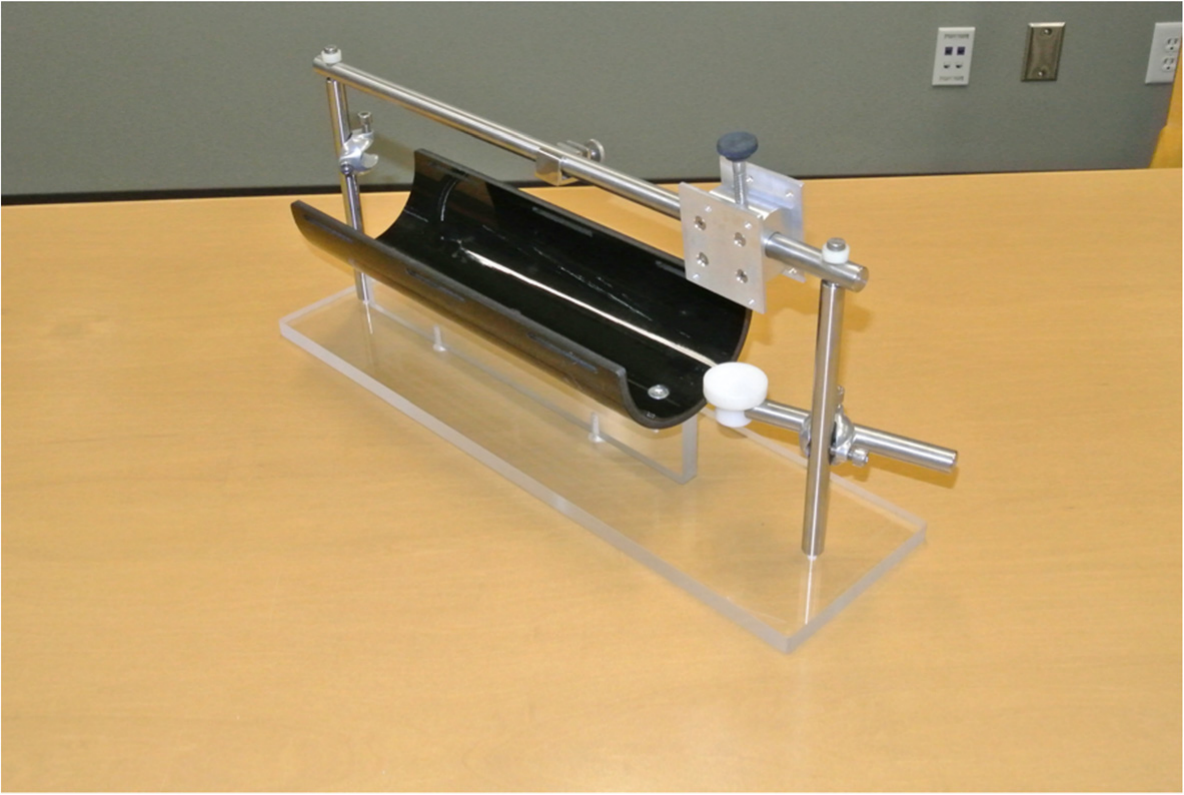
Customized body restraint

Based on the design described by Heaton et al., a custom body restraint device for the rat subjects was bioengineered (Metalworks Engineering Shop, University of Alberta, Edmonton, AB) [11]. Our body restraint apparatus consisted of a half-pipe (ABS-DWV IPEX Drainway) measuring 7.6 cm in diameter and 30 cm in length. Three Velcro® straps were then fastened across the top of the half-pipe for added restraint. A steel bar spanning across the half pipe provided a fixation point for the head implant as well as functioned to support the laser micrometers. Along the anterior portion of the half-pipe we added a circular platform to support the weight of the rat’s head while placed in the apparatus (Fig. 1).

#### Tracking whisker movement

Two pairs of photoelectric sensors (Rx-Laser Micrometer, Metralight Inc., San Mateo, Ca) were placed along each side of the subject’s face in order to track whisker movement. Thin tubing 1.5 mm in diameter was placed over a midline whisker on either side of the subject’s face to facilitate tracking by the laser micrometer. The laser micrometers were placed at 17° from the midline along each side of the face and this was considered parallel to the lateral surface of the face and positioned 10 mm from the origin of the tracked whisker on each side of the face.

The laser micrometer was comprised of an emitter, which produced a 780 nm wavelength light curtain, and a detector composed of a 28 mm linear array of 4000 charge-coupled devices (CCD scanline). A 5 cm vertical distance separated the emitter and detector, producing a laser curtain. Movement detected within the laser curtain sent a digital signal that could then be recorded. The laser micrometers were calibrated to avoid detection of objects less than 1 mm in size to prevent tracing of multiple whiskers. The calibrated laser curtain detected only the marked whisker.

#### Data acquisition

Whisker movement was elicited in each subject by providing a scented stimulus (chocolate milk). The laser micrometers themselves were connected to a 32-Channel Digital I/O Module (NI 9403, National Instruments, Dallas, Tx), which received digital output from the laser micrometers. The I/O module was connected to a PC through a CompactDAQ chassis (cDAQ-9174, National Instruments, Dallas, Tx). The I/O module acquired the laser micrometer signal at a sampling rate of 1 kHz. LabVIEW (LabVIEW Full Development System, National Instruments, Dallas, Tx) software was used as the interface for data acquisition.

### Surgical procedure

All subjects underwent both facial nerve surgery and head implantation surgery during the same anesthetic. Group 2 additionally received 1 h of BES following nerve repair while remaining anesthetized. All rats were first anesthetized with 3–4 % isoflurane. Subjects were then maintained under general anesthesia using 1.5 % isoflurane. Fur was then removed from the right side of the face and the top of the head using an electric shaver.

#### Facial nerve surgery

Facial nerve surgery was completed on the right side on all subjects. A small incision was made just inferior to the right ear bony prominence. Under microscopic visualization, the parotid gland was visualized, everted, and retracted out of the surgical field. Distal branches of the facial nerve were identified just inferior to the parotid bed. These were followed proximally until the main trunk of the facial nerve was identified. Once identified, the main trunk and upper and lower bifurcation of the facial nerve were carefully dissected. A single transection of the main trunk of the facial nerve was made using straight microscopic scissors; the cut nerve ends were then immediately repaired using a direct end-to-end technique. Using 9-0 sutures, four simple interrupted sutures were made within the proximal and distal epineural nerve endings. Care was taken to ensure proper nerve alignment.

#### Brief electrical stimulation

Along with facial nerve repair, animal subjects in group 2 received brief electrical stimulation. The stimulation protocol was adapted from one used by Gordon et al. in the sciatic nerve rat model [6]. Two silver Teflon coated wires were bared of insulation for 2–3 mm (AGT0510, W-P Instruments, Inc.). Following nerve repair, the first wire was looped around the proximal stump of the facial nerve. The second wire was imbedded into muscle tissue adjacent to the facial nerve, at a location just proximal to the first wire. The insulated wires were led to an isostim stimulator (A320D, W-P Instruments, Inc.) which delivered a 1.5 mA current in pulses of 100 microseconds in a continuous 20 Hz train for a period of 1 h. The adequacy of stimulation was verified by the presence of a right ear flutter. At the completion of stimulation, the wires were removed from the animal and the incision closed with interrupted 3-0 vicryl sutures.

#### Head implant surgery

**Fig. 2:**
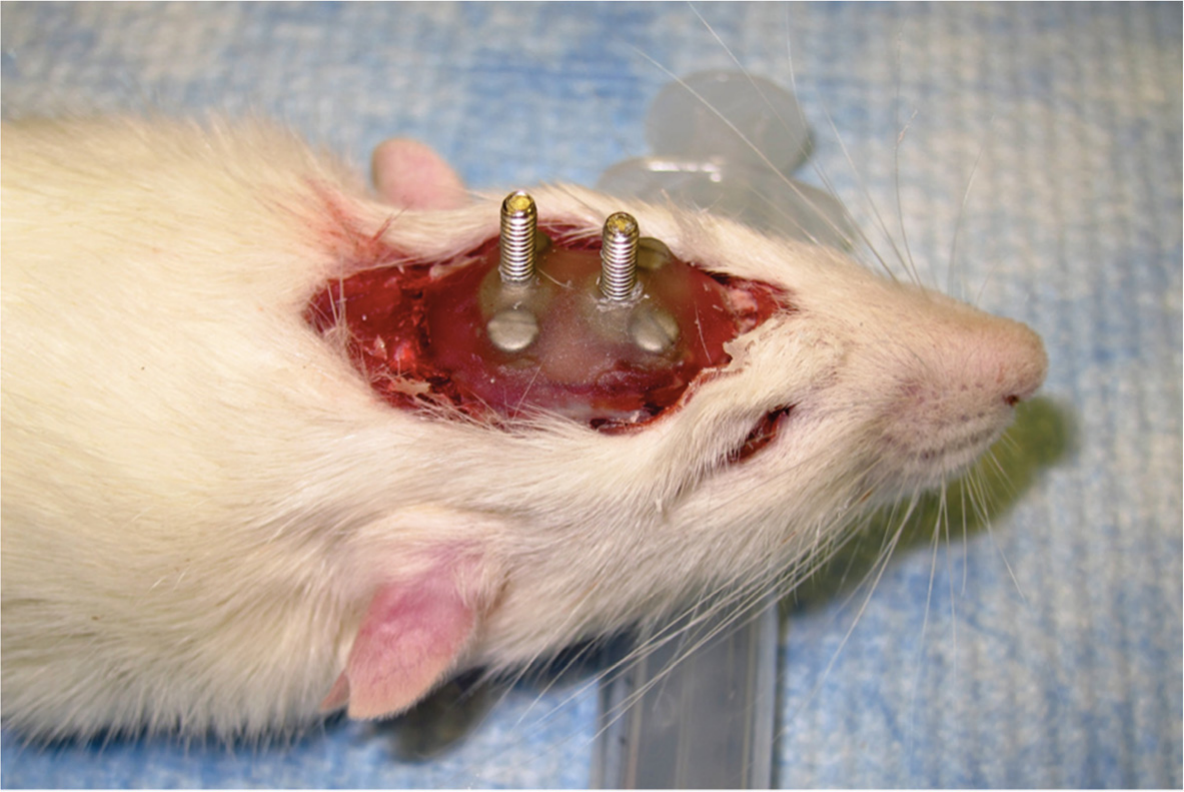
Acrylic helmet

Following the facial nerve procedure, head implant surgery was then completed without reversing the general anesthetic. A small incision was made using a 15-blade scalpel from the anterior to posterior margin of the cranium. Blunt dissection was employed to fully expose the underlying bony cranium. Using an electric drill, 4 holes were made in each quadrant of the skull approximately 15 mm apart from each other. 1.6 mm screws were then placed within each drill site (Fig. 2). Dry acrylic resin was then liquefied and placed onto the skull, covering the placed screws. Two larger 5 mm threaded screws were then inverted with the threads directed upwards into the acrylic before it solidified.

### Head fixation and body restraint

**Fig. 3:**
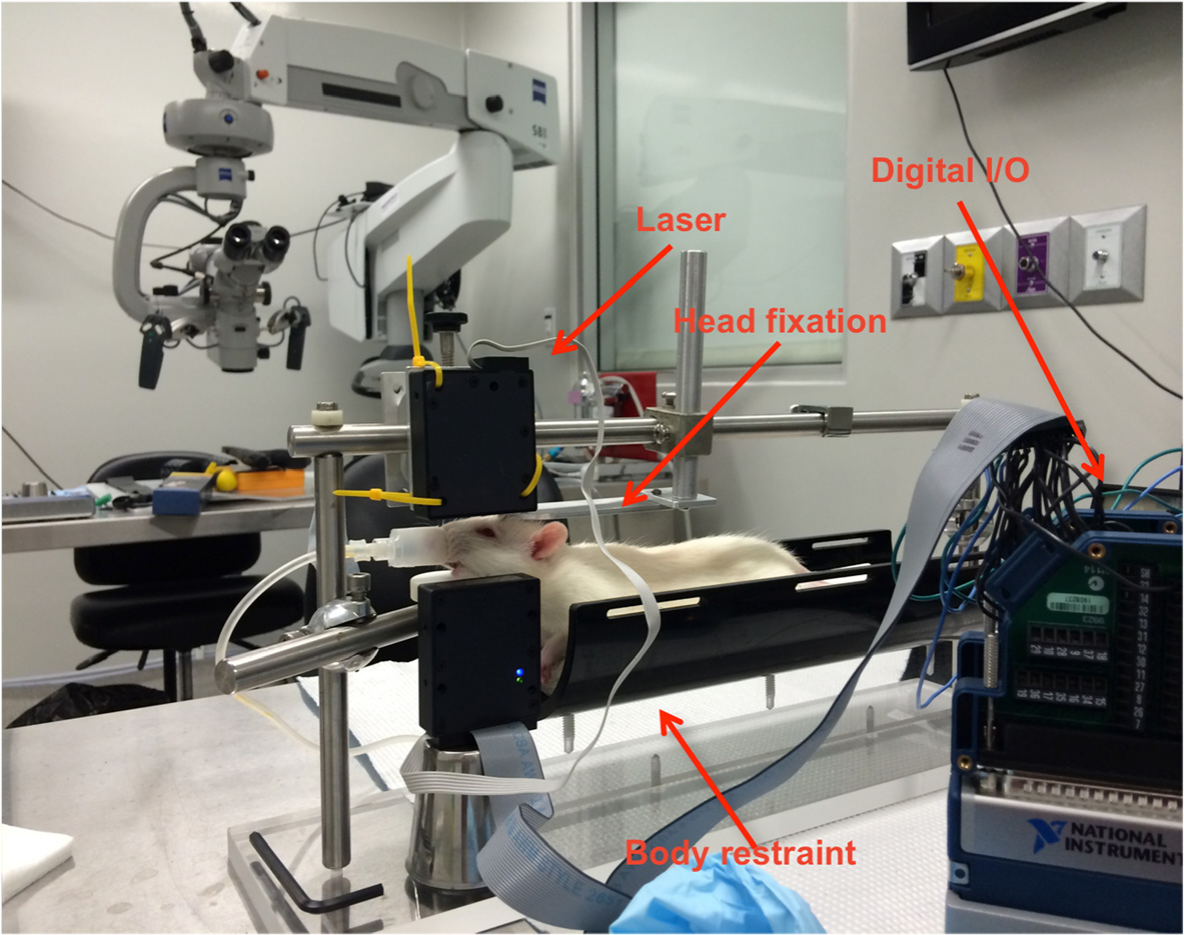
Whisking model

**Fig. 4:**
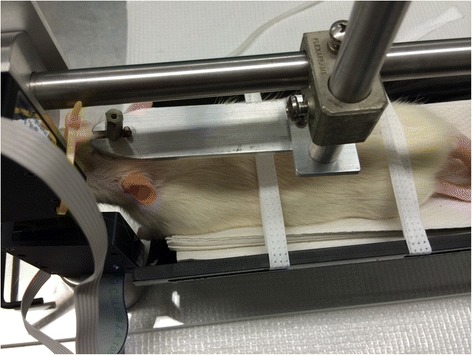
Head fixation

Two weeks prior to surgery, all animal subject were handled daily for conditioning. After surgery, all subjects were placed in body restraints daily for a week. At post-operative day 14, whisker measurements were started. Subjects were initially given dose low dose isoflurane and transported to the body restraint apparatus described in section 3.2 (Fig. 3). Here they underwent head fixation with bolts applied across the exposed threaded screws (Fig. 4). Whisker markers were then placed on either side of the rat’s face as described in section 3.3.

Once this was completed, a scented stimulus was introduced and recording started usually for a period of 5 min. The non-operative left side was used as the control for each subject. This procedure was completed for each rat at 2, 4, and 6 weeks post-operatively.

## Results

All animals tolerated the surgical procedure without perioperative complications. They exhibited normal cage behavior and did not lose weight. Three animals had problems with the head implantation device. In these animals, the device became loose at approximately week 4. This required an addition anesthetic with isoflurane and a new acrylic device to be made and fixed in place on the cranium. No animals had to be removed from the study.

**Fig. 5:**
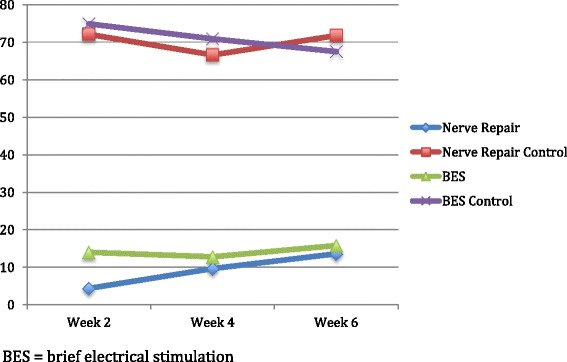
Whisking amplitude in degrees at 2, 4, and 6 weeks postoperatively. *BES* brief electrical stimulation

All animals experienced complete ipsilateral loss of whisking amplitude post-operatively. At week 2 the average amplitude observed for group 1 was 4.4° (Table 1). Showing a statistically significant improvement over group 1, the group 2 average was 14.0° at 2 weeks post-operatively (*p* = 0.0004). At week 4, group 1 showed improvement having an average of 9.7°, while group 2 remained relatively unchanged with an average of 12.8°. The week 6 results showed the greatest improvement from baseline for group 1. Group 1 had an average amplitudes of 13.63° at 6-weeks from surgery. Similarly, group 2 showed a slight increase in amplitude with an average of 15.84°. There was no statistically significant difference between the two groups at 4 and 6 weeks after facial nerve surgery (Fig. 5) (Table 2).

**Table 1:** Post-operative whisking amplitudes at week 2, 4, and 6

	Week 2 amplitude (degrees)	Week 4 amplitude (degrees)	Week 6 amplitude (degrees)
Nerve repair (group 1) Right side (operated)	4.4	9.7	13.63
Nerve repair (group 1) Left side (control)	72.1	66.6	71.8
BES (group 2) Right side (operated)	14.0	12.8	15.84
BES (group 2) Left side (control)	74.9	70.9	67.5

**Table 2:** Statistics for experimental groups at week 2, 4, and 6

	Week 2 amplitude (degrees)	Week 4 amplitude (degrees)	Week 6 amplitude (degrees)
Nerve repair (group 1) Right side (operated)	4.4 +/− 1.0	9.7 +/− 5.0	13.6 +/− 8.1
BES (group 2) Right side (operated)	14.0 +/− 6.6	12.8 +/− 8.3	15.8 +/− 10.9
*P*-value	0.039	0.515	0.779

## Discussion

Our animal study directly compared the facial nerve functional outcome in a group of rats receiving brief electrical stimulation following nerve transection and repair compared to those not receiving stimulation. Our results indicate a significant improvement in whisking amplitude in those animals receiving BES over those that did not in the early weeks following nerve surgery; however, by week 6 post-operatively, the difference between the two groups no longer bore statistical significance. Similarly, Nix et al. detected earlier and larger electromyographic signals in reinnervated rabbit soleus muscles with electrical stimulation after crush injury [12]. Our findings support conclusions made by these earlier rabbit studies, that electrical stimulation can accelerate early axonal regeneration and the rate of recovery of peripheral nerves.

Results of our study are consistent with other reports investigating the effects of electrical stimulation on peripheral nerve regeneration. Gordon et al. were able to demonstrate that electrical stimulation of the sciatic nerve in a rat model accelerated both axonal regeneration and the development of preferential motor reinnervation [6]. The authors also found that electrical stimulation of the sciatic nerve for 1 h was as effective in motor axonal regeneration as electrical stimulation for up to 2 weeks. The stimulation model we employed was based on the methodology described by Gordon et al. [6] Our results showed an initial acceleration in whisking amplitude in the stimulation group over the control group. However, by week 6 this difference had dissipated and both groups were found to have similar whisking measurements. Interestingly, Gordon et al. also found an initial acceleration in the number of motor neurons that regenerated into appropriate muscle in the animals that received electrical stimulation. However, by week 8 both groups showed similar motor neuron numbers [6]. Hadlock et al. also showed similar results in their 2010 rat facial nerve transection study. By week 11, the initial acceleration of whisking amplitude of the electrical stimulation rat group had equalized with the control group [2].

Gordon et al. have hypothesized that preferential motor reinnervation in a nerve injury model begins occurring at approximately 2 to 3 weeks following injury [6]. Prior to that moment, inappropriate sensory pathways are being created at the same rate as appropriate motor pathways. It appears that electrical stimulation is capable of starting preferential motor reinnervation at an earlier time point compared to non-stimulated nerves. Acceleration of preferential motor regeneration could contribute to counteracting the delay of nerve reinnervation pathways that are known to compromise functional outcome.

Although our study was not designed to detect synkinesis, the results of our study taken together with the findings of other researchers indicate the potential for acceleration of facial nerve function with electrical stimulation in animals. Although there are currently no human trials using BES following facial nerve injury, its application in the human clinical setting appears optimistic. Gordon et al. were able to demonstrate that patients receiving BES following carpal tunnel release surgery increased muscle reinnervation as early as 3 months following surgery [13]. Wong et al. demonstrated slight improvement in functional outcomes in humans receiving BES following digital nerve injury compared to a control group [14]. Rodents are also known to possess a greater ability to regenerate peripheral nerves and therefore modest animal findings may in fact indicate more significant potential results in humans. Future work will include corroborating our whisking findings facial muscle fiber count as well as facial motor neuron studies.

## Conclusion

In our study, we have shown that brief electrical stimulation of a rat facial nerve transection model accelerates whisker movement and therefore potentially facial nerve function. If facial nerve function is accelerated, brief electrical stimulation has the potential ability to counteract nerve reinnervation delays that are known to affect overall outcome. This has interesting clinical benefits and potential applications in human facial nerve injuries.

### Ethics approval

Prior to commencement of this study ethics approval was obtained from the University of Alberta Health Research Ethics Board.

## Abbreviations

ACUC: Animal Care and Use Committee
BES: brief electrical stimulation
HSLAS: Health Sciences Laboratory Animal Services
SMRI: Surgical Medical Research Institute
UAPWC: University Animal Policy and Welfare Committee
